# Variation of caesarean section rates in Palestinian governmental hospitals

**DOI:** 10.1186/s12884-022-05275-w

**Published:** 2022-12-16

**Authors:** Aisha Shalash, Yasmeen Wahdan, Hasan M. M. Alsalman, Ala’a Jamal Kamel Shehab, Tayseer Afifi, Hendia A. Nabaa, Iman Sarsour, Naheel Jarour, Alaa Hamed, Niveen M. E. Abu-Rmeileh

**Affiliations:** 1 Institute of Community and Public Health, Birzeit University, West Bank, State of Palestine; 2 School of Medicine, University of Limerick, Limerick, Ireland; 3 New Jericho Hospital, West Bank, State of Palestine; 4 Qalaqilia Hospital, West Bank, State of Palestine; 5 Faculty of Medicine, Islamic University of Gaza, Gaza, State of Palestine; 6 Continuing Education Department (PHC)/Salfit, Palestinian Ministry of Health(MOH), West Bank, State of Palestine; 7 Al Hilal Al Emarati Hospital, Gaza, State of Palestine; 8 Palestinian Board of Obs and Gyne, Al Bireh, State of Palestine

**Keywords:** Cesarean section, Caesarean section rate, Implementation research, Health system factors, Hospital factors, Medical indications

## Abstract

**Background:**

Globally, the increased use of cesarean sections has become prevalent in high-income and low and middle-income countries. In Palestine, the rate had risen from 20.3% in 2014 to 25.1% in 2018. We have rates as high as 35.8% in some governmental hospitals and some as low as 15%. This study aimed to understand better why there is a variation in cesarean rates in governmental hospitals that use the same guidelines.

**Methods:**

A qualitative and quantitative research approach was used. In-depth interviews were conducted with 27 specialists, obstetrics and gynecologists, and midwives in five government hospitals. The hospitals were selected based on the 2017 Annual Health Report reported cesarean section rates. The interview guide was created with the support of specialists and researchers and was piloted. Questions focused mainly on adherence to the obstetric guidelines and barriers to the use, sources of information, training for healthcare providers, the hospital system, and the factors that affect decision-making. Each hospital's delivery records for one month were analyzed to determine the reason for each cesarean section.

**Results:**

The results indicated that each governmental hospital at the system level had a different policy on cesarean sections. The National Guidelines were found to be interpreted differently among hospitals. One obstetrician-gynecologist decided on a cesarean section at high-rate hospitals, while low-rate hospitals used collective decision-making with empowered midwives. At the professional level, all hospitals urged the importance of a continuous training program to refresh the medical team knowledge, in-house training of new members joining the hospital, and discussion of cases subjective to obstetrician-gynecologists interpretations.

**Conclusion:**

Several institutional factors were identified to strengthen the implementation of the national obstetric guidelines. For example, encouraging collective decision-making between obstetrician-gynecologists and midwives, promoting the use of a second opinion, and mandatory training.

**Supplementary Information:**

The online version contains supplementary material available at 10.1186/s12884-022-05275-w.

## Background

The world faces an alarming increase in cesarean sections (C-sections) [1–4]. The C-section rate has almost doubled globally in the last 15 years. Previously experts estimated that 10—15% of births warrant a C-section delivery [2, 3]. But the warrant rates are not significant compared to saving lives in cases where vaginal deliveries would pose a complication (30). The C-section procedures were applied in more than 15% of births in 106 countries. In contrast, C-sections were used in less than 10% of births in 47 countries [3, 5].

The studies [2, 4] investigated the causes of in-country C-section disparities. For example, the rate is five times higher amongst the most affluent communities than those listed as the limited resources in low- and middle-income countries. In addition, C-sections use was significantly observed among low-risk deliveries at birth, especially among educated women. Likewise, they were more frequent in both Brazil and China in private facilities than governmental facilities and saw more planned C-section deliveries in rural populations [6, 7].

Clinical guidelines warrant a systematic approach to gynecology cases to ensure significant complications and variations are not made in the treatment [8]. There are many causes for the variation in the implementation of the guidelines. The guidelines are usually written in a complex language, making them hard to understand [8]. Also, they describe ideal situations which require more financial and human resources than are readily available. They call for continuous training by specialists, increasing the financial burden and workload on most hospitals [9]. Finally, the guidelines lack a clear scientific base regarding data sources or human sources responsible for creating them.

Studies showed the process of decision-making could impact C-section choice. This process affects by doctors' beliefs of what is supposed medical or medical indications, absences of collective decision between staff as to lack of cooperation among midwives [8] and gynecologists, and maternal requests, and it is a crucial part of women's involvement in the decision-making process [9].

According to the Palestinian Ministry of Health and the Palestinian Central Bureau of Statistics [10], in 2014, 20.3% of births in Palestine were by C-section, with 22.7% in the West Bank and 17.3% in the Gaza Strip. In 2016, 24.9% of births in Palestinian government hospitals were by C-section, compared to 25.5% in 2017. [10]. In 2017, some hospitals' rates reached 35.8%, while other hospitals' rates were 15% or less. This demonstrates a distinction between hospitals using the same procedures. This variation may exist for numerous reasons, including financial considerations and maternal requests [11]. Variation in rates between hospitals in the same sector, all of which adhere to the same protocol, indicates a peculiarity in the working conditions of the facilities in question [12, 13]. This variation can also be due to patient and doctor-related factors, hospital factors, and guideline implementation.

The first-ever study to look at C-section rates in Palestine was conducted in Gaza [13] and was limited due to a lack of statistics relating to women who gave birth in private hospitals or public hospitals in the West Bank. In addition, the study [13] did not include information surrounding C-section indications, which may assist our understanding as to why differences exist amongst same-sector hospitals. It also did not reflect health professional perspectives regarding the guidelines on why they might think there is a variation in C-section rates between hospitals in the West Bank and the Gaza strip.

This study is the exploratory stage of an implementation project to reduce C-section rates in the West Bank and the Gaza strip. This study aimed to understand why there is a variation in cesarean rates in governmental hospitals that use the same guidelines. The study also emphasized the barriers within governmental hospitals that prevent equal guidelines implementation.

## Methods

We used a qualitative and qualitative secondary data analysis approach to explore the variation in C-section rates among governmental hospitals in the West Bank and Gaza. The study is part of a more significant implementation study that aims to lower C-section rates in the country. In addition, we sought to explore healthcare providers' perceptions of the factors that influenced decision-making in governmental hospitals regarding C-sections.

The qualitative approach consisted of in-depth face-to-face interviews with health professionals, midwives, obstetricians and gynecologists (OB/GYNs) in labor wards in different governmental hospitals. Midwives are essential staff involved in the birthing process. They accompany women throughout the delivery and have a good assessment of their progress to advise and discuss with the specialists. The interview guide was developed in English, with the OB/GYNs in the research team, and the investigation of existing literature on the possibilities of the variation of C-section rates in hospitals with the same policies and guidelines. It was then translated into Arabic and back to English to check accuracy. Interviewers received training on qualitative methods and piloted the interview guide to ensure questions were understandable. The interview guide consisted of questions concerning participants' backgrounds, perceptions of vaginal and C-section deliveries, knowledge of women's delivery choices, national guidelines, and human resource training. The interview guide is available in Supplement 1.

The participants were chosen from hospitals that had high and low C-section rates. These hospitals were identified using the C-section rates stated in the Palestinian Ministry of Health Annual Report of 2017 [10]. Interviews were conducted with health professionals working the morning shift in the labor wards in each hospital on different days to ensure the participation of a more significant number of health professionals at the selected hospital. The morning shift includes a more significant number of staff and OB/GYNs. The interviews were done either in the doctor's or the head of the midwives office to ensure confidentiality and keep the participant from work distractions. Unfortunately, this was not achieved as some participants had to stop the interview several times to respond to emergency calls. The average time for the interviews was 45 min.

Interview transcription and analyses were run simultaneously while continuing the interviews in the selected hospitals. We stopped the interviews when we reached saturation and when similar answers were obtained. Three team members coded the data extracting themes and sub-themes using the transcription and notes taken during interviews. Thematic data analysis was done using Excel.

Two focus group discussions in a workshop format were conducted to discuss the study findings with the medical teams working in the studied hospitals. OB/GYNs, midwives, in addition to the director of the women's health department, hospital administration, and the committee involved in the guideline development, attended the workshops.

Quantitative secondary data analysis was done by requesting one month (October 2018) of deliveries from each hospital. The data collection sheet had maternal outcome, baby outcome, and mode of delivery. This can be found in Supplement 2. (The computer center within the Ministry of Health in the West Bank provided the OB/GYN notes). The doctor's notes contained all the information about induction, fluid, abdominal soft, dilation, and medical data such as drugs. We managed to extract the indication for C-section deliveries from the notes. In Gaza, patient files were not computerized, so the research team used paper files to extract the indication of a C-section delivery in each hospital for October 2018. Indications for C-sections were grouped into three categories: clearly stated in the guidelines, not clearly stated (which either allows for a second opinion or is based on the gynecologist's expertise), and not mentioned in the guidelines. Quantitative analysis was performed using SPSS 24.

The study was carried out in accordance with ethical guidelines of Birzeit University, Institute of Community and Public Health ethical review committee. Furthermore, informed consent to participate was obtained from all participants. After receiving informed consent from the participants, interviews were recorded and transcribed.

## Results

The research team conducted twenty-seven interviews in five West Bank and the Gaza strip hospitals between February and March 2019. We interviewed seven OB/GYNs and six midwives from three different hospitals in the West Bank and twelve OB/GYNs, one nurse, and one midwife from two hospitals in the Gaza strip. To give a better understanding of the included hospitals, Table 1 gives you a description of the hospitals included. Unfortunately, due to confidentiality, we cannot provide the names of the hospitals visited.

**Table 1:** Description of included hospitals

Hospital Number	Hospital Number 1	Hospital Number 2	Hospital Number 3	|Hospital Number 4	Hospital Number 5
C-Section Rate	High Rate	High Rate	High Rate	Low Rate	Low Rate
Territory	West Bank	West Bank	Gaza strip	West Bank	Gaza strip
Number of Gynecologists	5	3	38	5	10
Number of Residents	12–15	No residency program	29	14	12
Numbers of Midwives	15	16	83	29	35
Number of Nurses	-	-	71	-	68
Number of Anesthesiologists	-	3	35	3	5
Number of Operation Rooms in Hospital	-	3	4	6	2
Number of Fetal Monitoring (CTGs)	8	5	19	8	8
Number of Instruments Assisted in Births	4 forceps 6 vacuums	2 forceps 2 vacuums	5 forceps vacuum-not reported	1 forceps 2 vacuums	2 forceps 2 vacuums

There were senior and junior OB/GYNs as well as head midwives and midwives. Their job experience ranged from 1–25 years of experience. Table 2 contains a description of the characteristics of the participants.

**Table 2:** Description of participants

Participant Number	Job Position	Level of Education	Years of Experience	Hospital Number	C-section Rate	Territory
1	Senior (Department Head)	Gynecologist	10 years	Hospital number 1	high rate	West Bank
2	Senior	Gynecologist	2 years	Hospital number 1	high rate	West Bank
3	Midwife (Head of Midwifery)	Diploma in Midwifery	15 years	Hospital number 1	high rate	West Bank
4	Midwife	Bachelor of Midwifery	3 years	Hospital number 1	high rate	West Bank
5	Senior	Gynecologist	8 years	Hospital number 1	high rate	West Bank
6	Junior (third year)	Gynecology resident	resident	Hospital number 2	high rate	West Bank
7	Midwife (Head of Midwifery)	Diploma in Midwifery	13 years	Hospital number 2	high rate	West Bank
8	Midwife	Bachelor of Midwifery	1 year	Hospital number 2	high rate	West Bank
9	Senior	Gynecologist	2 years	Hospital number 2	high rate	West Bank
10	Senior (Department Head)	Gynecologist	25 years	Hospital number 3	high rate	Gaza strip
11	Midwife	Bachelor of Midwifery	6 years	Hospital number 3	high rate	Gaza strip
12	Midwife	Bachelor of Midwifery	6 years	Hospital number 3	high rate	Gaza strip
13	Senior	Gynecologist	4 years	Hospital number 3	high rate	Gaza strip
14	Senior	Gynecologist	12 years	Hospital number 3	high rate	Gaza strip
15	Senior	Gynecologist	4 years	Hospital number 4	low rate	West Bank
16	Senior	Gynecologist	2 years	Hospital number 4	low rate	West Bank
17	Midwife (Head of Midwifery)	Diploma in Midwifery	25 years	Hospital number 4	low rate	West Bank
18	Midwife	Bachelor of Midwifery	3 years	Hospital number 4	low rate	West Bank
19	Midwife	Bachelor of Midwifery	5 years	Hospital number 4	low rate	West Bank
20	Junior (second year)	Gynecology Resident	resident	Hospital number 4	low rate	West Bank
21	Midwife (Head of Midwifery)	Diploma in Midwifery	20 years	Hospital number 5	low rate	Gaza strip
22	Midwife	Bachelor of Midwifery	11 years	Hospital number 5	low rate	Gaza strip
23	Senior (Department Head)	Gynecologist	20 years	Hospital number 5	low rate	Gaza strip
24	Midwife	Bachelor of Midwifery	7 years	Hospital number 5	low rate	Gaza strip
25	Midwife	Bachelor of Midwifery	3.5 years	Hospital number 5	low rate	Gaza strip
26	Senior	Gynecologist	16 years	Hospital number 5	low rate	Gaza strip
27	Senior	Gynecologist	9 years	Hospital number 5	low rate	Gaza strip

Palestinian governmental hospitals' labor wards work follows a unified Palestinian Obstetrics Guideline. All the healthcare professionals interviewed found it beneficial for clarity and consistency in their work. But unfortunately, it was not entirely followed by all staff. From the interviews, we identified human resources and hospital factors as the main themes related to implementing the national guidelines and decision-making that would be considered a barrier associated with the variation of C-section rates in governmental hospitals.

### Implementation of guidelines

#### Human resources

The shortage of staff and work overload were critical factors in performing a C-section instead of a vaginal delivery. Vaginal delivery takes, on average, between 2–24 h from admission and delivery. Women from distant places or areas with checkpoints will stay in the hospital until they start active labor; C-sections take around 1 h between preparation and delivery. When there are many women with limited staff, women will be transferred to C-sections with no clear indications. In low C-section rate hospitals, even with staff shortages, they try to support vaginal deliveries because they have other supportive factors such as shared decision-making. Staff shortage was seen as a barrier to vaginal delivery in hospitals with low and high C-section rates. In addition, the lack of OB/GYNs does not allow for a second opinion or provide the right scientific discussion environment to reach the right decision. For example, three OB/GYNs are not enough to manage two shifts seven days per week.

"In this department, we have only three specialists/GYN

"We are so tired(laughing)when 20–25 women come in together, and we are only five midwives!" Midwife

Training and coaching are critical supports for staff as they learn to follow the guidelines. The medical teams mentioned several barriers to why the guidelines are not thoroughly followed. The Ministry of Health's training initially targeted the department head OB/GYN and the head midwife. The Ministry aimed to create trainers within each hospital that would then go back and train their staff. Each health care provider was given a copy of the guidelines. However, the staff working in the ward is high, and it wasn't easy to provide similar training to their other team members. As a result, the training was during brief periods, sporadic, and not comprehensive.

"The guidelines were delivered to every OB/GYN and midwife." OB/GYN

"Yes, it's available as hard and soft copy." OB/GYN

"There is no time to refer to the guidelines because of the workload." Midwife

"I don't know why we didn't receive training, probably because of time limits; we barely can complete our tasks during our shifts." Midwife

Head midwives and OB/GYNs trained their staff when the new guidelines were implemented, but new staff and residents did not receive formal training upon employment. Residents recall using books they used during their medical school training. When seeking employment, it was assumed that the guidelines were taught as part of the curriculum and that the staff and residents didn't need to be retrained. It was distinct that the team needed and desired to receive training, but time was limited, and the way they received training was not sufficient. Due to these barriers, they found the implementation of the guidelines difficult.

"No, we did not receive training on the guidelines (on employment), but sometimes we try to read certain topics to learn more (if needed)." Midwife

Although most of the staff interviewed use the guidelines, they could not see the difference between the older and newer versions, finding it unnecessary to update their information. They also indicated that some points were vague or unclear. Another barrier that came out was detachment or exclusion from the preparation process of the guidelines. Some OB/GYNs felt they should have been included in the preparation process. The OB/GYNs used the word "they" to refer to the committee members involved in guideline development to underestimate their experience and knowledge.

"They wrote the guidelines." OB/GYN

"There is not a noticeable difference between the old and new versions. There are a few changes. I didn't receive training on the new guidelines. Anyways, I was still studying the old version when it came out and read it myself." OB/GYN

"The guidelines were drawn up in the Palestinian Ministry of Health about ten years ago; it is incomplete because it is not this simple. These guidelines do not cover it (childbirth process)."OB/GYN

Those who found points vague or did not receive proper training preferred to use updated international sources continuously. They refer to evidence-based papers and universal guidelines such as the Green Top, issued by the Royal College of Obstetricians and Gynecologists [14]. In contrast, others refer to their medical school textbooks, which they used to obtain their degrees.

#### Hospital factors

One of the most significant health system barriers to decision-making related to delivery was hospital factors. Poor coordination between primary and secondary care within the Ministry of Health facilities poses a big challenge for the OB/GYNs and midwives. In addition, doctors have to deal with women without their medical history or information about their antenatal period, which poses a significant challenge for the medical team.

"The patient comes to us with a women's health booklet from the primary health care (as her only form of patient history). Each line has a different shape and is not clear. I cannot care for her or evaluate her using this booklet. These problems are due to the lack of connection between the primary health care system and government hospitals." Midwife

Also another factor was the influence of private hospitals on governmental hospitals. The most common reason for high C-section rates in governmental hospitals is that women have had a previous C-section, usually in a private hospital. There is a more significant financial benefit for private hospitals when performing a C-section than a vaginal delivery, so it is felt private hospitals encourage C-section deliveries even when not medically necessary. Maternal requests for your first C-section are not allowed in governmental hospitals. However, private hospitals promote C-section delivery with success. When wanting to deliver her second child, the same woman will go to a governmental hospital for a C-section to save money. The guidelines allow for flexibility in performing a C-section after having a previous C-section per the doctor's discretion. A higher C-section rate is typically in areas with more private hospitals reported by the Ministry of Health annual report, and lower in regions lacking private hospitals in the West Bank and the Gaza strip.

"Private hospitals increase the C-section rates for us. They perform a C-section in the first pregnancy, so most of the next births will be a cesarean, which they come to government hospitals because of the cost." OB/GYN

Some think the guidelines were prepared to be used in ideal conditions, not in Palestinian hospitals. In addition, the working environment regarding caseload, staff, beds, and lack of equipment and tools prevent the guidelines' full compliance.

"They give us the guidelines, but there is no equipment, no tools, no place or environment to apply them." OB/GYN

"We are trying to make the guidelines appropriate for our work, but it needs a quieter environment." Midwife

Some hospitals have policies that do not adhere to the guidelines or allow OB/GYN interpretation to accommodate these challenges. For example, the guidelines state that a previous C-section does not indicate a C-section. Still, from the interviews in high C-section rate hospitals, OB/GYNs considered it an indication. On the other hand, with the low C-section rate hospitals, they give women who have had a previous C-section a chance for vaginal delivery before performing one.

"If everything is normal and the baby is in a cephalic position, we give her a trial period for a vaginal delivery." Midwife

"In the past, we tried to give them a chance for vaginal delivery (previous C-section), but now, no! We mostly go for a C-section." OB/GYN

One OB/GYN had described the guidelines as similar to a traffic light: green- clear statement to go for C-section delivery; red- clear statement not to go for C-section delivery; orange- this depends on OB/GYN's experience, consultation, and other sources utilized. Variation in C-section rates arises from the imbalance between decisions based on experiences only, based on the guidelines only, and based on both.

### Decision making

Leadership is critical in decision-making related to the mode of delivery. Some hospitals reported classic, hierarchical power structures. The head OB/GYN holds the highest power, then the OB/GYNs, residents, head midwives, midwives, and nurses, if available in departments such as in Gaza. With this structure, an OB/GYN makes the decision, and in some hospitals, one OB/GYN decides without having a second opinion. This was seen in high C-section rate hospitals. Midwives who spent most of their time with the patients were not consulted in decision-making.

"One OB/GYN can decide to do a cesarean section." Midwife

"It is clear one OB/GYN can decide to do a cesarean section. This depends on the situation, but the OB/GYN can make the decision (without a second opinion)." OB/GYN

"We cannot influence the OB/GYN's decisions." Midwife

In low C-section rate hospitals, collective decision-making involving the residents and the midwives and getting second opinions differed from high C-section rate hospitals. If a resident presented an argument for or against C-section, their opinion was valued and considered. Midwives felt their thoughts were valued in helping OB/GYN make decisions.

"There are always discussions between the OB/GYN and the resident in deciding to go for a C-section." Midwife

"In other settings, midwives cannot influence the OB/GYNs, but roles are distributed between the team, and mutual respect is observed." Midwife

"The decisions are shared by the OB/GYN, senior OB/GYN, and midwives responsible for following up with women." OB/GYN

In addition to the guidelines, OB/GYN's scientific qualifications and experience affected the decision-making process. For example, it was clear that the mentor, hospital, or university where the OB/GYN got their qualifications significantly influenced decisions. This was common among all interviewed OB/GYNs.

"Decisions sometimes have to be made based on qualifications and experience when having to act fast."OB/GYN

An interesting observation was regarding the gender of the OB/GYN. Low C-section rate hospitals have more female OB/GYNs. It is unclear whether female OB/GYNs are more patient or sympathize with the women, but it is an observation.

"We have female OB/GYNs; they have more patience with the women." Midwife

### Hospital records

The indications for C-sections recorded in the hospital records are presented in Table 3. The most common C-section indication in high-rate hospitals is women with a previous C-section or two or more C-sections, followed by a breech presentation and fetal distress. In low-rate hospitals, the most common indications are women who have had previous two or more C-sections, a previous one C-section, followed by a breech presentation.

**Table 3:** Indication of Caesarean Section according to the Ministry of Health Guidelines

Hospital Number	Hospital 1	Hospital 2	Hospital 3	Hospital 4	Hospital 5
C-Section Rate	High Rate	High Rate	High Rate	Low Rate	Low Rate
Territory	West Bank	West Bank	Gaza strip	West Bank	Gaza strip
**1-Indication of C-section in the guidelines (clear)**					
Previous C-Sect. 2 +	27.20%	40.30%	32.90%	25.00%	28.70%
Breech	9.50%	9.70%	19.40%	16.70%	9.50%
Fetal distress	1.80%	3.20%	1.80%	17.90%	11.00%
**2-Indication of C-section in the guidelines (not clear)**					
Previous 1 C-section	39.1%	29.00%	22.40%	11.90%	17.0%
Placenta Previa	5.30%	0.00%	0.30%	3.60%	1.00%
Failure of Induction/ Failed Progress	3.60%	6.50%	5.40%	3.60%	8.40%
In-vitro Fertilization	3.00%	1.60%	5.00%	1.20%	6.00%
**3-Indication of C-section in the guidelines (not mentioned)**					
Twins -Triplet	2.40%	3.20%	2.00%	10.70%	6.10%
4-Other indications:	8.40%	6.40%	9.80%	10.80%	8.81%
Total	100%	100%	100%	100%	100%

Comparing the high-rate and low-rate hospitals, we noticed that the breech presentation and previous two or more C-section rates matched and followed the guidelines to deal with these medical indications. Some non-medical indications for C-sections mentioned that OB/GYNs use to justify a C-section included in vitro fertilization, old primigravida, uncooperative patient, high blood pressure, uncontrolled diabetes mellitus, big baby, and fibroids. These terms were mentioned mainly in high-rate C-section hospitals, but big baby or old primigravida was noted in both settings.

After the interviews, two workshops were conducted to discuss the study findings with the medical teams working in the study's hospitals. Obstetricians, midwives, the women's health department director, hospital administration, and OB/GYNs involved in developing the Palestinian National Guidelines attended the workshops. There was an agreement on the main finding regarding the need to continue staff training and find the best modalities while addressing the workload and infrastructure limitations. There were debates and lengthy discussions on updating the guidelines, so it is more apparent regarding some of the indicators involved in C-section delivery decision-making. All participants appreciated having a proper referral system between primary and secondary care. Most often, hospitals receive women with no information about their antenatal care and conditions. All also agreed on the private sector's influence in encouraging C-section deliveries and that there should be clear regulations within the private sector.

## Discussion

Our study opened the door to discussion, although all governmental hospitals should use the same national obstetric guidelines. There is a variation in cesarean section rates among different hospitals. It was essential to understand why there was a variation and if appropriate. We found that hospitals followed the guidelines to the best of their abilities. Still, a variance in staff training and coaching, the department's leadership style, and ambiguity of specific indications of C-section in the guidelines allowed for differences in C-section rates among the hospitals.

All hospital labor departments included in the study suffered from a shortage of staff and excessive workloads. Head specialists and head midwives received training from the Ministry of Health on the national obstetric guidelines and were required to train their staff. The training of trainers can be a valuable model for training staff Yolsal, Bulut [15] if trainers are given the proper time and resources to train their needed staff [16]. Unfortunately, head staff were not given the time because of patient load; no efficient time was available for training. Also, staff hired after the implementation of guidelines were not given orientation. However, assumptions were made to be aware of the new policies and act accordingly. Continuous education will allow for more successful implementation of the guidelines and guidelines [17]. To combat the lack of time for trainers, the implementation of continuous online education has become an effective and acceptable form of training [18]. Allowing the staff to view the training on their own time, with interactive videos, and allowing for group discussions makes online training almost equivalent to in-house training [19].

One of the main differences between the high and low C-section rate hospitals was the leadership style. Low-rate hospitals empowered their midwives and junior medical staff, involved them in their decision-making, and made most C-section decisions as a team and not individually. Midwife-led models, midwife continuity of care, and mandatory second opinions are known interventions that significantly affect C-section rates in high-class countries and could be tested in low- and middle-income countries [1]. For example, a hospital in Sweden saw a decline in C-section deliveries by adopting a 9-item list of changes in delivery practices. One of the changes was ensuring that the OB/GYN, midwife, and nurse worked together to discuss each woman and any complications they might face [20]. Low-rate hospitals applied these interventions to help them with unnecessary C-section operations.

Another observation of low C-section rate hospitals was that they had more female OB/GYNs than males. However, a systematic review and meta-analysis of delivering physicians and C-section rates found that women were 25% less likely to perform a C-section, especially without medical indication than men [21]. As a result, the guideline's revision team didn't include a female OB/GYN. Instead, the women included were nurses and associate professors, with all the male contributors being OB/GYNs. The last edit was in 2015; we hope that contributions from more OB/GYNs will help them take ownership of the guidelines for future modifications.

Lastly, the national labor and obstetric guidelines have left some indications of C-sections to be ambiguous and left up to the OB/GYN. We find that it would be necessary for these indications to be revised to be more transparent in the guidelines. Guidelines developed by the implementation group are more likely to be implemented correctly [22]. In an observational study looking at the compliance of general practitioners to guideline use, the study showed that when the recommendations were ambiguous and non-specific, they were followed 36% of the time compared to 67% when recommendations were explicit [23]. Guidelines should include information on using them and contain specific and easy words [24]. They give concrete details on particular situations and how they can be used to address them and are more likely to be implemented [25]. The guidelines should also be exact and include complex information about all possibilities [26].

The secondary data from charts and hospital documentation was fragmented. The OB/GYN notes were not complete and were designed to be used only by the medical staff, not for decision-making or analysis, making assessing and understanding current practice challenging. Applying the Multidisciplinary Quality Assurance Programme and collecting quality information will help ensure quality care is given [27]. A slight modification in the system could help the team group all deliveries according to the Robson Classification and help understand the management process of the whole child birthing process, including C-section decision-making. The classification will categorize women attending the labor ward into one out of ten categories based on their gestational weeks, fetus position, and the number of fetuses. The Robson Classification system will allow for more information on the patients and their fetal outcomes to analyze the use of C-Sects. [18, 27]. This categorization will help decide whether the C-sections are not being overused and whether the rates are not considered high but necessary. The Robson classification implementation in hospitals will allow medical teams to evaluate delivery management processes and provide a standardized method for reporting and comparison between hospitals, regions, and countries [28].

## Conclusion

This study is an exploration phase of implementing the obstetric guidelines and assessing the barriers in decision-making regarding C-section births. At this stage, we could identify a lack of leadership, proper training, and organizational drivers to limit the implementation of the obstetric guidelines. There is a need for frequent updates of the guidelines based on recent evidence and continuous feedback from the medical staff. It will be essential to find creative ways to train and coach the current guidelines with the limited resources and work overload. Providing a supportive environment is crucial in properly implementing and addressing insufficient infrastructure and health staff. A reasonable C-section rate is essential, but it should not be at the expense of women or babies. All evidence should be collected to ensure the best care quality.

### Strengths and limitations

This study provided labor ward staff perspective on the reasons behind the C-sections rates in their hospitals and the challenges facing the implementation of the national obstetric guidelines. The research team was able to visit hospitals both in the West Bank and the Gaza strip, making the research national instead of based in one territory. However, the analysis was limited to C-section cases in one month and did not include vaginal delivery, which may mask each hospital's workload. Another challenge faced by this study was working with the medical team in the labor ward to find the best time to talk freely. The medical team is constantly under pressure and has a very high load. The research team tried to overcome this limitation by visiting the hospital several times and interviewing the staff during the morning shift when more staff were available.

## Supplementary Information

**Additional file 1.**

**Additional file 2.**

## Data Availability

The datasets used or analyzed during the current study are available from the corresponding author upon reasonable request.

## Abbreviations

C-Section: Caesarean Section
OB/GYN: Obstetricians and Gynecologists
