# Supplementary material for: Variation of caesarean section rates in Palestinian governmental hospitals
Source: BMC Pregnancy Childbirth. 2022 Dec 16;22:943. doi: 10.1186/s12884-022-05275-w (PMC9756638; doi:10.1186/s12884-022-05275-w)
Supplement: Supplementary file 1 — Additional file 1. [file 12884_2022_5275_MOESM1_ESM.docx]

**Health Providers Interview Guide**

Oral Consent

This is a study to understand variation in CS rates in governmental hospitals. We would like to discuss with you how we could explain these variations in numbers. Your participation is voluntary and you can stop answering any question or the interview at any time. The information collected will be treated with confidentiality and your name will not appear in the final report. We would like to record the interview, if you do not mind. The recorded interview will be erased after completing the research. If you give us your oral consent, we would like to start.

هذه دراسة لفهم التباين في معدلات العمليات القيصرية في المستشفيات الحكومية الفلسطينية. نود أن نناقش معك هذه  الاختلافات في البيانات لفهم اسبابها. مشاركتك طوعية، يمكنك التوقف عن الإجابة على أي سؤال أو المقابلة في أي وقت. سيتم التعامل مع المعلومات التي يتم جمعها بسرية ولن يظهر اسمك في التقرير النهائي. لاغراض بحثية، نود تسجيل المقابلة. ستكون المقابلة المسجلة متاحة فقط لفريق البحث، و سيتم  حذف المقابلة المسجلة بعد الانتهاء من البحث. ان كنت موافقا على المشاركة ، سنبدا المقابلة.

Interviewer signature that oral consent was given:_________________ Date:___________

**Health Providers Interview Guide**

Could you give us some information about your training, how long have you been working in this hospital and other hospitals? What is your current position in the hospital? .

1. هل يمكن أن تعطينا بعض المعلومات حول تدريبك ، كم من الوقت كنت تعمل في هذا المستشفى والمستشفيات الأخرى؟ ما هو وضعك الحالي في المستشفى؟

Protocol questions:

1. أسئلة البروتوكول:

- Is there a protocol for labor and cesarean section delivery you follow?
- ھل ھﻧﺎك ﺑروﺗوﮐول للولادة و اﻟﻌﻣﻟﯾﺎت اﻟﻘﯾﺻرﯾﺔ اﻟﺗﻲ ﺗﺗﺑﻌﮭﺎ ؟
- Tell me about the protocol. (if answer yes to the first question)
- أخبرني عن البروتوكول. (إذا كان الجواب نعم على السؤال الأول)
- Do you know of any other protocol that is used?
- هل تعرف أي بروتوكول آخر يستخدم؟
- Do you follow the protocol? Yes or no why not?
- هل تتبع البروتوكول؟ نعم أو لا؟ لم لا؟
- Do you use it all the time? (the protocol) yes…..if no why not?
- هل تستخدمها طوال الوقت؟ (البروتوكول) نعم ... ..إذا الجواب لا لماذا لا؟
- Do you think the protocol you use needs to be updated?
- هل تعتقد أن البروتوكول الذي تستخدمه يحتاج إلى تحديث؟
  - If yes, What do you think needs to be updated?
  - إذا كانت الإجابة بنعم ، ما الذي يجب أن يتم تحديثه؟
- Did you receive any training on the protocol you use? If yes ask, if no… why no training given?
- هل تلقيت أي تدريب على البروتوكول الذي تستخدمه؟ إذا كانت الإجابة بنعم ، إذا لم يكن الأمر كذلك ... فلماذا لم يتم إعطاء تدريب؟
  - Do you think it was enough training? Why or why not?
  - هل تعتقد أن التدريب كان كافيا؟ لماذا او لماذا لا لم يكن كافيا
- Do you think the training for the protocol is appropriate for your working conditions?
- هل تعتقد أن التدريب على البروتوكول مناسب لظروف العمل الخاصة بك؟
  - Why or why not?
  - لماذا أو لماذا لا؟
- Did any of your colleagues receive such training?
- • هل تلقى أي من زملائك مثل هذا التدريب؟

Now we will ask about Caesarean sections and the decision making process

الآن سوف نسأل عن العمليات القيصرية وعملية صنع القرار

1. What is the decision making process for CS?العمليات القيصرية ما هي عملية صنع القرار في
   1. Who makes the decision to conduct a CS? من يتخذ قرارًا بإجراء العمليات القيصرية
   2. On what basis is the decision to conduct a CS made? على أي أساس يتم اتخاذ قرار إجراء العمليات القيصرية
   3. Do the interns or residents follow the decision if it is not based on the MoH protocol? هل يتبع المتدربون أو المقيمون القرار إذا لم يكن مبنياً على بروتوكول وزارة الصحة
2. Which mode of delivery would you select for the following medical indication? ما هي طريقة الولادة التي تختارها لدلائل الطبية التالية
   1. Repeated C-section ( history of 1 or more C-sections) why?
   2. Placenta Previa
   3. Breech presentation when not in labor
   4. Breech presentation in labor
   5. Fetal distress
   6. What kind of fetal monitoring do you have? Continuous or intermittent?
   7. Approach towards a women with a previous one C-section
   8. Twins gestations-- When the second twin is not cephalic
   9. Threshold for big baby
   10. Methods used for induction of labor for prolonged pregnancy and for those with fetal or maternal indications for termination of pregnancy
   11. Age of the women as an indication
3. What is your opinion on the following non- medical indication for CS? ما هو رأيك في الدلائل غير الطبية التالية للولادة القيصريةـ
   1. Precious baby, like prolonged history of infertility, IVF pregnancy
   2. Women preference/ choice (maternal request)
4. Would you like to add anything else in regards to the CS rate in your hospital?

هل ترغب في إضافة أي شيء آخر فيما يتعلق بمعدل العمليات القيصرية في المستشفى الخاص بك ؟
