# Supplementary material for: Variation of caesarean section rates in Palestinian governmental hospitals
Source: BMC Pregnancy Childbirth. 2022 Dec 16;22:943. doi: 10.1186/s12884-022-05275-w (PMC9756638; doi:10.1186/s12884-022-05275-w)
Supplement: Supplementary file 2 — Additional file 2. [file 12884_2022_5275_MOESM2_ESM.docx]

Supplement 2

**Maternal and Baby Outcomes Sheet**

CS No: Date:

Patient ID:

Type of CS

1. Emergency 2. Elective

Indications (written in file):

________________________________________________________________________________________________________________________________________________________________________________________________________________________________________________________________________________________________

Maternal Outcomes:

Hospital stay: Duration:______________

Injury: 1. Yes 2. No Type:_________________

Blood transfusions: 1. Yes 2. No Why?_________________

Fetal Outcomes:

Apgar score at 1minute ____________ .

Apgar score at 5 minutes ____________.

Admission to NICU: 1. Yes 2. No
